# Comparative genomic analysis of vertebrate mitochondrial reveals a differential of rearrangements rate between taxonomic class

**DOI:** 10.1038/s41598-022-09512-2

**Published:** 2022-03-31

**Authors:** Paula Montaña-Lozano, Manuela Moreno-Carmona, Mauricio Ochoa-Capera, Natalia S. Medina, Jeffrey L. Boore, Carlos F. Prada

**Affiliations:** 1 Grupo de Investigación de Biología y Ecología de Artrópodos, Facultad de Ciencias, Universidad del Tolima, Ibague, Colombia; 2 Providence St. Joseph Health and Institute for Systems Biology, 401 Terry Avenue N, Seattle, WA 98109 USA

**Keywords:** Computational biology and bioinformatics, Evolution, Genetics

## Abstract

Vertebrate mitochondrial genomes have been extensively studied for genetic and evolutionary purposes, these are normally believed to be extremely conserved, however, different cases of gene rearrangements have been reported. To verify the level of rearrangement and the mitogenome evolution, we performed a comparative genomic analysis of the 2831 vertebrate mitochondrial genomes representing 12 classes available in the NCBI database. Using a combination of bioinformatics methods, we determined there is a high number of errors in the annotation of mitochondrial genes, especially in tRNAs. We determined there is a large variation in the proportion of rearrangements per gene and per taxonomic class, with higher values observed in Actinopteri, Amphibia and Reptilia. We highlight that these are results for currently available vertebrate sequences, so an increase in sequence representativeness in some groups may alter the rearrangement rates, so in a few years it would be interesting to see if these rates are maintained or altered with the new mitogenome sequences. In addition, within each vertebrate class, different patterns in rearrangement proportion with distinct hotspots in the mitochondrial genome were found. We also determined that there are eleven convergence events in gene rearrangement, nine of which are new reports to the scientific community.

## Introduction

Vertebrate mitochondrial genomes are circular, typically 14–20 kbp, and contain genes for 13 proteins (atp6, atp8, cob, cox1–3, nad1–6, nad4L), 2 ribosomal RNA (rRNAs; rrnS, rrnL), 22 transfers RNA and two non-coding regions: L-strand origin replication (OL) and control region (OH or D-loop) which is known to contain controlling elements for replication and transcription^1–3^. Gene rearrangement is one of the most studied features for animal mitochondrial genomes (mtDNAs)^4^ and generally the order of genes on the mitochondrial genomes is considered to be conserved^5^. However, it has been reported some rearrangements include gene transposition, gene loss and gene duplication^6^. These events have often been modeled by a process of tandem duplication followed by random gene losses (TDRL) which is the most frequently invoked model to explain the diversity of gene rearrangements in metazoan mitogenomes^6–8^.

Due to advances in genomic databases (RefSeq database from NCBI)^9^, it has become possible to compare hundreds of vertebrate mitochondrial genomes from various taxonomic lineages, showing that gene order can vary far more than previously recognized^1,10,11^. Although the vertebrate ancestral mitochondrial gene arrangement is found conserved^1^, some rearrangements have long been noted among lamprey^12^, some species of fish^11,13^, amphibians^14,15^, some species of lizards^16,17^, snakes^18^, tuatara^19^, crocodilians^20^, birds^21^ and marsupial mammals^22^; most of these rearrangements involve genes flanking one or both of the two origins of replication, sites where gene duplications that have been proposed to mediate translocations may be especially common^1,8,22–26^.

Differences in size in some vertebrate mitochondrial genomes are due to nucleotide insertion or deletion events, mainly in the hypervariable domain of control region^27–31^ and some cases to gene duplication and deletion generally in tRNAs^3^. Rearrangements of mitochondrial genes can have profound functional implications on gene expression and genome replication, can be correlated with genomic variation, aspects of physiology, molecular mechanism, life history, or genomic evolutionary processes^2,32^.

The aim of this study is to systematically compare the rate of mitochondrial gene rearrangements in each of the taxonomic orders of vertebrates and to identify possible convergence events that occurred in the evolution of this important animal group.

## Results

### Gene annotation errors in the vertebrate mitochondrial genome

There are a total of 104,904 genes annotated in the 2831 vertebrate mitochondrial genomes found in the NCBI database, including 178 gene duplications detected by the presence of an identical copy of such gene. A total of 1951 cases are annotated to be in arrangements differing from the ancestral vertebrate mitochondrial gene order (Supplementary Table S1). Of all these reorganizations, 389 were identified as gene annotation errors, an error percentage of 20%. Of these, tRNAs have the highest level of annotations errors (94.6%) with the highest values in *trnE* (15.3%) and *trnP* (14.6%), 4.3% are errors in rRNA genes, represented mostly by errors in *rrnS* (3%), and 1.1% of the annotation errors in coding genes are only in the case of *nad6* gene. Actinopteri, Reptilia, Aves, and Mammalia with 123, 89, 77, and 68 genes with annotation errors, respectively (Table 1).

**Table 1:** Number and distribution of analyzed mitochondrial genomes with summary of types of deletions, duplications, inversions, translocations that have been reported in GenBank correctly or as errors.

Class	No. orders	No. species	No. of different genomes within the order	Genes differing in rearrangement	Numer confirmed	Dup	Del	Inv-tra	Numer refuted	% of reorganization
Myxini	1	2	A	0	0	0	0	0	0	0
Petromyzonti	1	6	1 + A	6	6	0	0	6	0	2.70
Elasmobranchii	12	74	A	2	0	0	0	0	2	0
Holocephali	1	5	A	0	0	0	0	0	0	0
Cladistii	1	2	A	0	0	0	0	0	0	0
Actinopteri	59	1259	60 + A	751	628	25	9	594	123	1.61
Coelacanthi	1	1	A	0	0	0	0	0	0	0
Dipneusti	1	3	A	0	0	0	0	0	0	0
Amphibia	3	241	34 + A	292	262	55	4	203	30	3.27
Reptilia	6	314	26 + A	564	475	30	4	441	89	4.85
Aves	28	620	15*	149	72	68	4	0	77	0.65
Mammalia	29	304	2 + A	187	119	0	0	119	68	1.66
		2831	–	1951	1562	178	21	1363	389	

Taxa includes taxonomic classes analyzed without reorganizations: Myxini, Elasmobranchii, Holocephali, Cladistii, Coelacanthi and Dipneusti.

*Dupli.* duplications, *Dele.* deletions, *Inver.* inversions, *Trans.* translocations.

*The order has no species with the ancestral gene order. A: Ancestral architecture, in some taxonomic groups in addition to the different architectures (with rearrangements) organisms with the ancestral organization can also be found.

### Rearrangement level in the vertebrate mitochondrial genome

**Figure 1:**
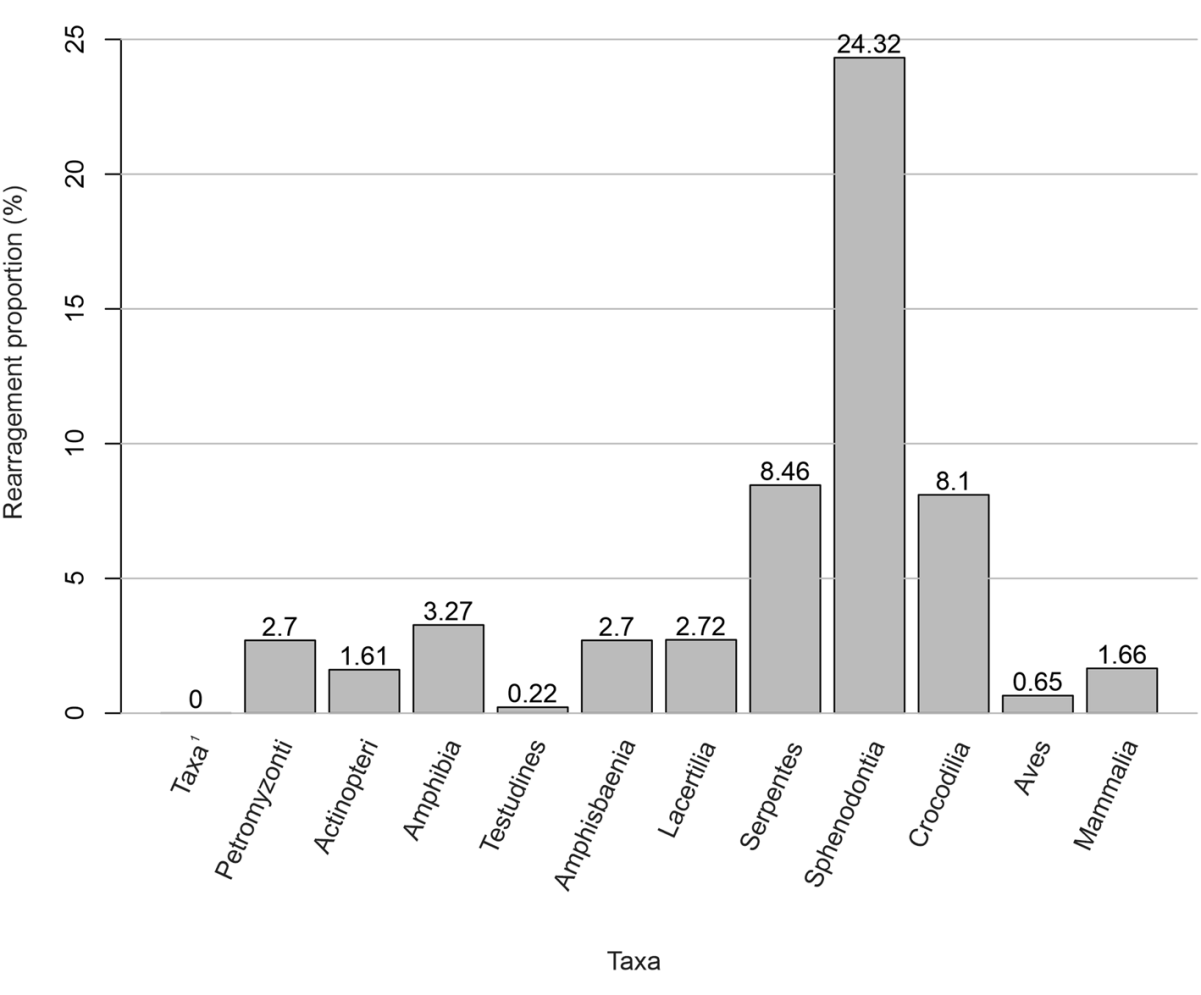
Gene rearrangement proportion values for each of the vertebrate classes sampled. Taxa^1^ includes taxonomic classes analyzed without reorganizations: Myxini, Elasmobranchii, Holocephali, Cladistii, Coelacanthi and Dipneusti.

In this study a total of 1562 reorganizations in the vertebrate mitochondrial genome were confirmed. Our results show that 36% (1020 of 2831) of the mitogenomes (present in 6 of the 12 class) have at least one rearrangement (Table 1). The present work revealed that the species with the most rearrangements were in the classes Actinopteri (628), followed by other taxa such as Amphibia (262), and suborders Serpentes (285) and Lacertilia (112) for class Reptilia (Table 1). However, when calculating the rearrangements rate by class, these vary from one to another; showing that the classes with low rearrangement rate (> 0 to 5%) are order Testudines (0.22%), order Amphisbaenia (2.70%) and order Lacertilia (2.72%) for class Reptilia, Amphibia (3.27%), Petromyzonti (2.70%), Mammalia (1.66%), Actinopteri (1.61%); and Aves (0.65%). The only taxonomic groups with vertebrate ancestral order were Myxini, Elasmobranchii, Holocephali, Cladistii, Coelacanthi and Dipneusti (Table 1 and Fig. 1).

Within some taxonomic classes, there are orders wherein all sampled species retain the ancestral order and others with gene rearrangements. For example, in the Actinopteri class, 34 of the 59 taxonomic orders have all sampled species retaining the ancestral vertebrate mitochondrial gene arrangement; the other orders have species with one or more rearrangements. The order Saccopharyngiformes (belonging to Actinopteri class), has many species (72.8%) with rearranged mtDNAs in comparison with other orders of the same class as Anguilliformes (5.1%); observing gene rearrangements in only 4 analyzed genomes. Within the Reptilia class, the Sphenodontia (24.3%) with a widely reorganized single species, followed by Serpentes and Crocodilia orders have higher proportions of reorganization (8.7% and 8.1%, respectively) compared to Amphisbaenia (2.7%), Lacertilia (2.7%) or Testudines (0.2%). Anura order (belonging to the Amphibia class), presents a major proportion of reorganization (4.8%) in comparison with Caudata (0.78%) or Gymnophiona (1.30%) orders (Fig. 1).

On the other hand, our results show that the most frequent rearrangements in vertebrates are inversions/translocations with 87.3% (1363/1562), followed by duplications with 11.4% (178/1562) and deletions with 1.3% (21/1562). However, in some classes, certain events predominate as in the case of Aves, where 94.4% (68/72) of the rearrangements are duplications and 5.6% (4/72) are deletions (there are no confirmed inversions/translocations in this class). In the case of Mammalia, only inversions/translocations were confirmed (Table 1). In addition, of all rearrangements, 85.3% (1332/1562) are of the tRNA genes, mainly associated with inversions/translocations (92%) (Supplementary Table S1).

**Figure 2:**
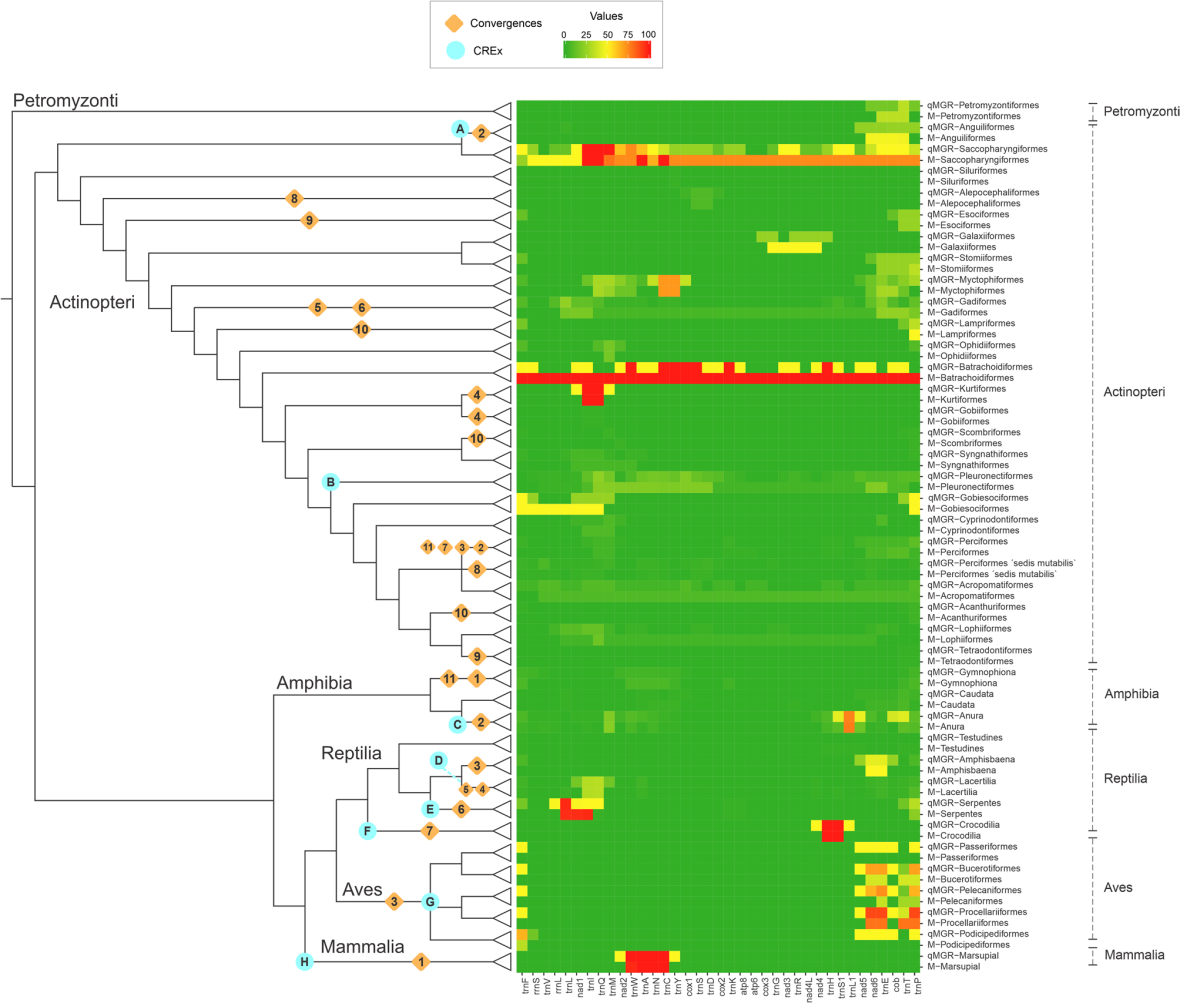
Heat map of gene rearrangement analysis among vertebrate classes. Phylogenetic relationships are as interpreted in Amemiya et al.^84^. Dark green colors show a low proportion of change and red colors show a high number of rearrangement events for each of the individual genes within each taxonomic order that exhibited rearrangements in mitochondrial sequences. Orange diamonds show the number of convergences detected in that taxonomic order; aquamarine blue circles indicate that a CREx representation was performed for that taxonomic order.

The results when quantifying the proportion of rearrangements with qMGR analysis in each gene by taxonomic order was different from our manual analysis. For example, within the class Actinopteri, our analyses confirm that most or all of the genes present a high proportion of gene rearrangements. In the taxa Batrachoidiformes, where only one species was available for analysis, rearrangements were found in 100% of its genes, while in Saccopharyngiformes (4 species analyzed, which were available at NCBI) 72.8% of the genes showed rearrangements. This percentage differs significantly compared to the qMGR result of 37.8% (Fig. 2). In this sense, the most rearrangements in vertebrate mitochondrial genome were observed mainly in tRNA genes, concentrating in certain clusters such as *trnL1, nad1, trnI, -trnQ, trnM; trnW, -trnA, -trnN, -trnC* and *trnH, trnS, trnL2, nad5, -nad6, -trnE, cob, trnT, -trnP* (Fig. 2). Of the 1562 genes with confirmed rearrangements, 103, 101, 145, 70, and 58 are present in the *trnL1, nad1, trnI, -trnQ, trnM* region, respectively; while 55, 56, 56 and 60 are observed in *trnW, -trnA, -trnN, -trnC*; 38, 44, 108, 30, 70, 80, 50, 81 and 85 are in *trnH, trnS, trnL2, nad5, -nad6, -trnE, cob, trnT, -trnP*. In contrast, it can be observed that within the vertebrate mitogenome there are relatively conserved blocks, as is the case of *cox2, trnK, atp8, atp6, cox3, trnG, nad3, trnR, nad4L, nad4* (9, 13, 11, 10, 9, 11, 10, 12, 10, and 12 rearrangements, respectively) (Fig. 2). Due to a lot of vertebrate mitochondrial genome (about 40%) presents a loss (partially or completely) of D-loop región, this was not taken into account for this analysis.

However, it can be observed that each taxonomic order in vertebrates, presents rearrangement proportions in different regions. For example, while in most taxonomic orders, the rearrangements are concentrated in adjacent genes of the control region, high rearrangements proportions in *trnI, -trnQ, trnM* and adjacent genes are shown in Pleuronectiformes, Myctophiformes (Actinopteri class) and Serpentes; Crocodilia in *nad4, trnH, trnS, trnL2*. Also, Marsupialia and mentioned taxonomic orders of Actinopteri class, share rearrangements proportions in *trnW, -trnA, -trnN, -trnC* (Supplementary Table S2, Fig. 2).

On the other hand, of the 178 duplications present in vertebrate mitochondrial genome, are concentrated mainly in tRNA genes with the 83.7% (149/178), observed in the *-nad6. -trnE, cob, trnT, -trnP* region (19, 23, 22, 29, and 35 copies of each gene respectively) and of the *trnM* gene (35 copies of the single gene; a single copy per mitogenome). The taxonomic class with the highest number of duplicated genes is Aves with 38.2% (68/178) followed by Amphibia with 30.9% (55/178) of all gene duplications detected in vertebrates. Most of the duplications observed in amphibians occurred in *trnM* gene (23 copies), all observed in Neobatrachia species of the order Anura. Another characteristic observed in Aves was the presence of duplicated pseudogenes (ineffective copies).

In this taxonomic group, 42 of these copies were observed, half of them (21) correspond to pseudogenes of *cob*; mainly observed in the order Pelecaniformes. In this same taxonomic order, it was also observed copies in the form of pseudogenes of *-nad6, -trnE, trnT and -trnP* (6, 2, 7, and 5, respectively). Although it is common to find a single additional copy (duplicate) of one or two genes, in certain genomes more than two presumably effective copies of the same gene are observed; in the case of *Cnemaspis limi* (Reptilia, Squamata) that contains four copies of the *trnA* gene. Similarly, tandem duplications of complete mitochondrial regions are observed, as in the case of *Aeluroscalabotes felinus* (Reptilia, Squamata) with 53 genes in total (18,974 bp) and in *Breviceps adspersus* (Amphibia, Anura) with 51 genes (28,757 bp) (Supplementary Table S1).

Of the 21 deletions that we founded, they also are concentrated in the tRNA genes (19/21), mainly in *trnT, -trnP* region with 3 and 6 deletions, respectively. The most frequent taxonomic group of deletions is Actinopteri with 9 deletions, mainly in the -*trnP* gene with 5 deletions (Supplementary Table S1).

### Gene arrangement convergence in the vertebrate mitochondrial genome

Our results indicate that within the 1020 reorganized mitogenomes (with respect to the order of the ancestral genes of vertebrate), 138 different genetic architectures were identified; 11 of these are grouped in convergences in the gene arrangement observed in 764 species (Table 2). The remaining 127 are unique genetic arrangements in vertebrates, as in the case of *Phrynocephalus przewalskii* (Squamata, Reptilia), with duplications of the *trnF* and -*trnP* genes, and inversions/translocations in *trnQ* and in the second copy of -*trnP* gene (Supplementary Table S1).

**Table 2:** Gene arrangement: convergence in the mitochondrial genome of Vertebrata.

Convergence	Taxonomic level	Order	N. genome
Convergence 1 (-*trnA, -trnC, trnW, -trnN*)	*Siphonops annulatus*	Gymnophiona (Amphibia)	1
		Marsupialia (Mammalia)	29
Convergence 2 (*cob, trnT, -nad6, -trnE)*	*Coloconger cadenati*	Anguiliformes (Actinopteri)	14
	*Ariosoma shiroanago*		
	*Paraconger notialis*		
	*Conger japonicus*		
	*Congriscus sp.*		
	*Heteroconger hassi*		
	*Derichthys serpentinus*		
	*Nessorhamphus ingolfianus*		
	*Cynoponticus ferox*		
	*Muraenesox bagio*		
	*Facciolella oxyrhyncha*		
	*Hoplunnis punctata*		
	*Nettastoma parviceps*		
	*Ophisurus macrorhynchos*		
	*Chaenocephalus aceratus*	Perciformes (Actinopteri)	3
	*Chionodraco hamatus*		
	*Notothenia coriiceps*		
	*Aneides flavipunctatus*	Caudata (Amphibia)	2
	*Stereochilus marginatus*		
Convergence 3 (*trnT, -trnP, -nad6, -trnE)*	*Pagothenia borchgrevinki*	Perciformes (Actinopteri)	1
	*Rhineura floridana*	Amphisbaenia (Reptilia)	1
		Aves	591
Convergence 4 (-*trnQ, trnI, trnM)*	*Kurtus gulliveri*	Kurtiformes (Actinopteri)	1
	*Ponticola kessleri*	Gobiiformes (Actinopteri)	1
	*Brookesia decaryi*	Lacertilia (Reptilia)	29
	*Chamaeleo africanus*		
	*Chamaeleo arabicus*		
	*Chamaeleo calcaricarens*		
	*Chamaeleo calyptratus*		
	*Chamaeleo chamaeleon*		
	*Chamaeleo dilepis*		
	*Chamaeleo monachus*		
	*Chamaeleo zeylanicus*		
	*Furcifer oustaleti*		
	*Kinyongia fischeri*		
	*Trioceros melleri*		
	*Acanthosaura armata*		
	*Acanthosaura lepidogaster*		
	*Chlamydosaurus kingii*		
	*Hydrosaurus amboinensis*		
	*Leiolepis boehmei*		
	*Leiolepis guttata*		
	*Phrynocephalus albolineatus*		
	*Phrynocephalus axillaris*		
	*Phrynocephalus grumgrzimailoi*		
	*Phrynocephalus guinanensis*		
	*Phrynocephalus helioscopus*		
	*Phrynocephalus mystaceus*		
	*Phrynocephalus putjatai*		
	*Pogona vitticeps*		
	*Pseudotrapelus sinaitus*		
	*Uromastyx benti*		
	*Xenagama taylori*		
Convergence 5 (-*nad6, cob, trnT, -trnP, -trnE*)	*Cetonurus globiceps*	Gadiformes (Actinopteri)	3
	*Coelorinchus kishinouyei*		
	*Ventrifossa garmani*		
	*Uroplatus fimbriatus*	Lacertilia (Reptilia)	1
Convergence 6 (*nad1, trnI, trnL)*	*Squalogadus modificatus*	Gadiformes (Actinopteri)	1
	*Trachyrincus murrayi*		1
		Alethinophidia-Serpentes (Reptilia)	57
Convergence 7 (*trnS, trnH*)	*Aulorhynchus flavidus*	Perciformes (Actinopteri)	1
		Crocodylia (Reptilia)	18
Convergence 8 (*trnD, -trnS)*	*Normichthys operosus*	Alepocephaliformes (Actinopteri)	1
	*Ambassis gymnocephalus*	Perciformes ‘sedis mutabilis’ (Actinopteri)	1
Convergence 9 (*cob, -trnP, trnT)*	*Dallia pectoralis*	Esociformes (Actinopteri)	1
	*Rudarius ercodes*	Tetraodontiformes (Actinopteri)	1
Convergence 10 (-*trnP* deletion)	*Trichiurus japonicus*	Scombriformes (Actinopteri)	1
	*Hapalogenys analis*	Acanthuriformes (Actinopteri)	1
	*Lampris guttatus*	Lampriformes (Actinopteri)	1
Convergence 11 (-*trnP* duplication)	*Clinocottus analis*	Perciformes (Actinopteri)	1
	*Boulengerula taitana*	Gymnophiona (Amphibia)	1
		Total	764

Our results show that different classes and orders have different genomic architectures, all the sampled species within Aves share a reorganization from the vertebrate ancestral order, but relatively conserved within this taxonomic class. Only 29 species of the 620 analyzed have any type of rearrangement with respect to the Aves ancestral order (see CREX analysis). Among order Saccopharyngiformes (Actinopteri), there are three different architectures in the four analyzed species, while in other taxonomic groups they are more conserved; for example, within the class Mammalia, all marsupials (29 mt genomes) share a single rearrangement from the vertebrate ancestral order (Table 1).

**Figure 3:**
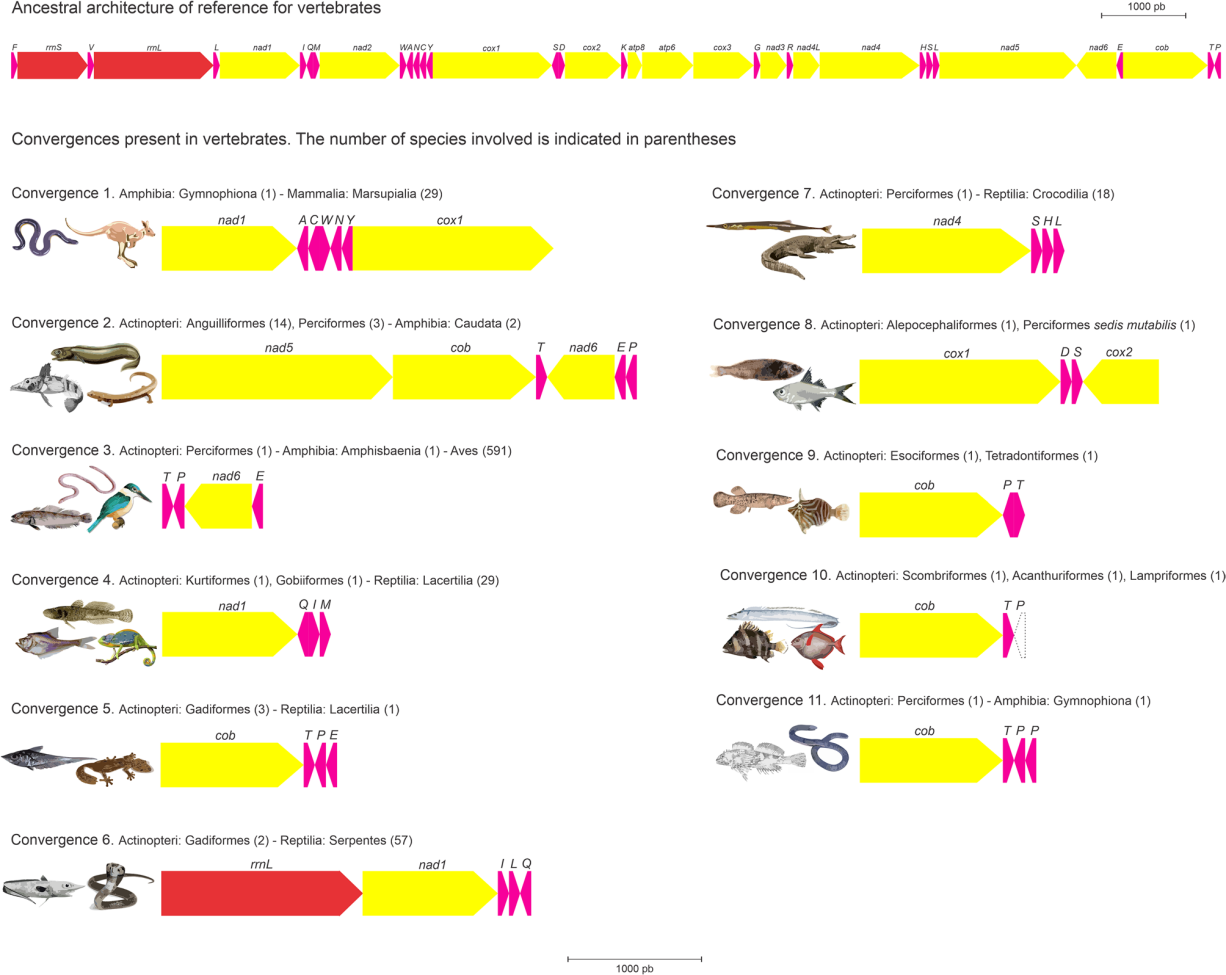
Evolutionary convergence of mitochondrial gene order rearrangements. This shows only the subsets that are rearranged; all other genes share the ancestral arrangement. Arrows show transcriptional orientation. tRNA-encoding genes are labeled with the one-letter code for the corresponding amino acid. In parentheses are the number of species involved in the convergence.

In the convergences (11) four of them (8–11) are between orders belonging to the same taxonomic class of fish, in addition, 10 and 11 are convergences in deletion and duplication events of a gene, respectively. All cases of convergence between different taxonomic orders are represented in Fig. 3.

### Analysis of gene order with CREX and TreeREx

CREX analysis was done with the purpose of determining a putative ancestral gene order for the taxonomic orders that presented more rearrangements, here are the scenarios of events that had to occur to produce a change between the ancestral order to the order of the current genomes (See Fig. S1). In many cases, a modest rearrangement from the ancestral vertebrate gene order occurred at the base of a large group of organisms and is shared broadly within that group, including: (1) a reverse transposition of *cob, trnT, -trnP* in Anguiliformes (Actinopteri); (2) a reverse transposition of -*trnQ* and -*trnC, -trnY*, a reverse transposition of -*trnS, trnD, cox2*, *trnK*, and a reversal of the region of *cox2 to trnH* in Pleuronectiformes (Actinopteri); (3) a transposition of *trnL2* Anura (Amphibia); (4) a reverse transposition of *nad1*, *trnI* genes, a reversal of the region *trnL2* to *nad1*, and a reversal of the region *cox2* to *trnH* in the infraorder Alethiniphidia (Serpentes, Reptilia); (5) a transposition of *trnS* in class Reptilia, order Crocodilia; (6) a transposition of the region *cob*, *trnT, -trnP* in Aves; and (7) a TDRL event for the region *trnW, -trnA, -trnN, -trnC, -trnY* in Marsupialia (Mammalia).

On the other hand, TreeREx is a useful algorithm for assigning rearrangements to the edges of a given phylogenetic tree, with which we reconstruct the ancestral genetic orders at the interior nodes of the most rearranged orders and identify a significant number of transpositions, reverse transpositions, inversions, and TDRL events (Supplementary Fig. S2). In Saccopharyngiformes (Actinopteri), TreeREx detected that most nodes are inconsistent and that the most common event is TDRL (Fig. S2A). In Myctophiformes and Pleuronectiformes (Actinopteri), TreeREx detected that most nodes are consistent; the most common events being transpositions (Fig. S2B) and TDRL, respectively (Fig. S2C). In Amphibia (Anura), TreeREx detected those nodes are mostly consistent, with transpositions being the most common event causing reorganizations in this order (Fig. S2D). In Lacertilia (Reptilia), TreeREx detected all the nodes are consistent and the most common event is the inversions (Fig. S2E).

## Discussion

### Gene annotation errors in vertebrate mitochondrial genome

Due to the advance of sequencing techniques in the last two decades, hundreds of mitochondrial genomes of different taxonomic groups of vertebrates have been deposited in the NCBI database and then subsequently curated into RefSeq files. Despite this, different authors have reported a significant number of gene annotation errors, most of which can be corroborated by basic bioinformatics tools^22,33–35^. One source of these errors may be from software that provides automated gene annotation^34^, but some misannotations stem from simply failing to note the proper orientation of a gene or an erroneous error of naming. Errors are also sometimes perpetuated by presuming that previously made annotations are correct and then following this for a newly sequenced mtDNA, the so-called “percolation of errors”^22^.

Prada and Boore^22^ reported that 36.3% of mammalian mitochondrial genomes obtained from the NCBI database analyzed had annotation errors. Our results, like those observed in mammals^22^, show that tRNA genes are more susceptible to errors in gene notation (94.6%) than other mitochondrial genes, with a higher error percentage in certain taxonomic groups (Actinopteri, Reptilia, and Aves). Popadin et al.^36^ suggest performing verification of tRNA gene annotations manually to ensure a higher level of accuracy in annotation, although it could also be done through a combination of semi-automated bioinformatics techniques^23^ where the curator would play an important role in detecting these errors.

A large number of studies on human mtDNA contain errors, a level so high that geneticists could be drawing incorrect conclusions in population and evolutionary studies^37^, so since the 2000s different authors already recommended greater controls by both journals and individual scientists, which we still recommend given that the errors are still persistent. Due to the large number of mitochondrial genomes being reported annually in the database, the probability that these errors in gene notation will continue to spread is high, so curation and generating broad-scale data-quality evaluations remain scarce of the data by the scientific community is recommended^38–40^. NCBI is also encouraged to implement elementary error checking mechanisms when promoting a submitted sequence into their RefSeq database as described previously^22,33^. Another possible source of gene annotation errors in mitochondrial genome is the technical differences between sequencing platforms. In this case, NGS is significantly cheaper, quicker and is more accurate and reliable than Sanger sequencing; however, the absence of the sequencing source in several mitochondrial genome database files makes it difficult to associate annotation errors with a particular sequencing methodology.

### Genome rearrangements and vertebrate mitochondrial genome evolution

Traditionally, vertebrate mitochondria have been considered to have a "conserved gene order” from primitive vertebrates (fish) to primates^2,41,42^. However, in some lineages, there is a variation in the rate of its reorganization. For example, within vertebrates, gene rearrangements have been found for some species of lizards, amphibians, fish, crocodilians, snakes, tuatara, and lamprey^1,11,15,18,19,43,44^. Nevertheless, in these studies, it is not identified which taxonomic groups are the most reorganized within them, or what is the differential reorganization rate among them.

Our results show a differential reorganization proportion within and between taxonomic classes, confirming that Reptilia, Amphibia, Petromyzonti (Lamprey), Mammalia, Actinopteri and Aves, present a proportion between 24.32% and 0.22%, in order of highest to lowest. For example, within the Reptilia class, we see a great variability in the proportion of rearrangements, where the order Sphenodontia with only one species (Sphenodon punctantus) presents the highest rate in vertebrates (24.3%), since a large part of its mitochondrial genes are rearranged. Different particularities in the mitochondrial genome of this species have already been reported^19,45^ including different factors involving light-strand replicational errors in the tandem duplication of genic regions^46^, molecular selection processes within the cells^47^ or adaptive processes to convergent habitats^48^; which possibly could be connected to evolutionary history of each species, although a lot of studies need to be held to correlate those causes^49^.

Crocodylia and snakes also have high proportions of rearrangements compared to other taxonomic groups, mainly explained by the fact that there are no species with the gene order considered ancestral. All Crocodylia have the order *trnS-trnH-trnL* (derived from an *trnS* transposition from the ancestral order of vertebrates), results consistent with those reported previously^1,50,51^; observing a unique genetic architecture for all this group (Alligatoridae and Crocodylidae).

Similarly, most snakes have the order *nad1, trnI, trnL,-trnQ, trnM* (derived from a reverse transposition and reversal of the ancestral order of vertebrates), which differs from the Viperidae with the order *nad1, trnI, -trnP, trnL, -trnQ, trnM* (derived from a *trnP* translocation), similar results with those reported previously^1,51,52^. However, our results show that the two species of snakes belonging to the Typhlopidae family (*Amerotyphlops reticulatus* and *Indotyphlops braminus*), present the ancestral order of vertebrates and not that of other snakes, consistent with an earlier report^53^.

In contrast, within the suborder Lacertilia (Reptilia), we observe that some taxonomic families such as Agamidae, Chamaelonidae and some species of the family Gekkonidae, present a higher rate of reorganization than others families such as Iguanidae or Lacertidae that retain the ancestral vertebrate order^51,54^.

Amphibians have been reported to be a more conservative group compared to reptiles^1^. However, our results show that within amphibians, the Anura order (124 species analyzed) is more reorganized, with a total of 103 species showing at least one reorganization event and only 21 with the ancestral vertebrate order. Of these reorganized species, most (86) share different gene orders, and the rest have unique architectures (18). Xia et al.^6^ presents the *cob, trnL, trnT, -trnP* gene order as an extensive reorganization in anurans (Neobatrachia), our results confirm this, and that the Anura mitochondrial genome presents greater variability in terms of genetic order than in comparison with other amphibians.

Previous studies have postulated that the genetic order *nad6-trnT-trnE-cob-trnP* is common among lampreys^1,55^, known to be the earliest diverged vertebrates with a time of divergence inferred to be 550 mya^56^. Although our results confirm the presence of this reorganization in two species of lampreys (*Lethenteron camtschaticum* and *Petromyzon marinus*) in the family Petromyzontidae, the three remaining species of this family with a reported mtDNA gene arrangement and the one species in the Geotriidae family retain the vertebrate ancestral gene order, so it is not correct to assert that it is an ancestral character of all lampreys.

According to Satoh et al.^11^, most of the fishes had the typical gene order widely shared among vertebrate mt genomes, noting that only 14% (35/250) of these species observed have at least one rearrangement, a percentage over three times as high (4.1%; 52/1255) as that presented by Gong et al.^57^. Although our results show a relatively low proportion, like those previously published, some taxonomic orders such as Anguilliformes, Saccopharyngiformes, Myctophiformes, Gadiformes, Batrachoidiformes, Pleuronectiformes, Perciformes, and Perciformes (and some listed as sedis mutabilis) are highly reorganized in comparison with other orders in fish. Many of such gene order are unique to a specific taxon, but some are shared polyphyletically between distantly related species.

Our results confirm that the majority of species within the class Aves share the *cob, trnT, -trnP, -nad6, -trnE* rearrangement from the ancestral vertebrate arrangement as described in early work^58^. In addition to this, multiple independent rearrangements have occurred in some species of birds, including genetic duplications of different tRNA-encoding genes (*-trnE, trnT, -trnP)* and cob pseudogenes as has been previously reported^10,59^. This work clarifies that these duplications are concentrated in certain taxonomic groups such as the family Ardeidae (Pelecaniformes), Procellariiformes and Suliformes, marine aquatic birds with great diving capacity, suggesting this gene order is the ancestral pattern within these birds and persisted in most lineages perhaps through concerted evolution^59^. According to Gibb et al.^60^, it is very possible that in species of birds of different orders, they have hidden duplications in the genome that also include the control region and cannot be observed because the genome assembly programs for short sequencing reads artifactually collapse these regions. Nevertheless, further physiological and molecular analyses are necessary to assess the potential selective advantages of the mitogenome duplications^61^.

The existence of actual gene deletions in mitogenomes is widely questioned^62^, in this study we found only 21 cases, which we propose could be associated mainly to sequencing or assembly errors, as is the case of the nad6 gene of the mitogenome of antarctic fish (Notothenioidei: Perciformes); in the first instance Papetti et al.^63^ proposed the loss of the gene, and later, the authors Zhuang and Cheng^64^ found and characterized it within the control region, which according to them is difficult to sequence with some methods. This last statement about the difficulty of sequencing the control region^65^ could explain why the largest number of deletions we found are in *trnT* and *-trnP* due to their proximity to this area of the genome.

Gene rearrangements in vertebrate mitogenomes have been explained mainly by two models, the Tandem Duplication and Random Loss Model^24^ and the Recombination Model^66^. The TDRL model has been found to explain most vertebrate gene rearrangements; where new gene orders result from the random deletion of one of the redundant pairs of paralogs produced by a tandem duplication. The gene that is deleted is presumed to accumulate random mutations that disrupt normal function and create a pseudogene that is selected and eventually lost from the genome. Therefore, it is difficult to trace which steps preserved functional genes and which DNA segments degenerated into pseudogenes or intergenic spacers^24^. On the other hand, the apparent total absence of recombination activity in the animal mt genomic system would suppress gene rearrangements and thus lead to a low incidence of rearrangement events, based on the assumption of absence of recombination in mt genomes, mt gene rearrangements have generally been mostly interpreted by the TDRL model, however, recent evidence of recombination in the animal mt genome urges reconsideration of other modes of recombination-mediated duplication^67–70^.

There are also two other models, the tandem duplication and non-random loss (TDNL) model and tRNA mispriming, which is used to explain mainly reorganization events in invertebrates^71,72^. In addition to the alternative mechanisms that account for simpler reorganizations including inversion^54,73^.

### Convergence in a hotspot of gene rearrangement

Our results, as found in previous analysis, have shown that the mitochondrial genome of vertebrates, in certain taxonomic groups, have a considerable proportion of reorganization. Most of these rearrangements involve tRNA genes, *nad5* has been observed for smaller studies^1,3,24,26,32,54,74^. These regions that are prone to rearrangements within the mitochondrial genome are commonly referred to as "hot spots" for rearrangement, which makes the likelihood of genomes from species that are not closely related converging on the same gene rearrangement high^23,24^. In this work, the identification of three “hot spots” (*trnL, nad1, trnI, -trnQ, trnM*; *trnW, -trnA, -trnN, -trnC* and -*nad6, -trnE, cob, trnT, -trnP*) in the mitochondrial genome of vertebrates in certain lineages. The *trnW, -trnA, -trnN, -trnC, -trnY* region, the cluster of five mitochondrial tRNA genes and the OL (replication origin of light strand) among them, has been reported as one of the most important hotspots for gene order rearrangements by TDRL^22^. These rearrangements involved translocations and insertions, which have been found in many vertebrate groups. Many previous studies have indicated that OL was possibly involved in processes of gene rearrangements^24,54^, mutation gradients^75^ and asymmetric nucleotide composition bias^76^.

In 2008, Kurabayashi, et al.^67^ reported that for frogs of the family Mantellidae the control region had the potential to cause rearrangements of genes adjacent to it. Likewise, it has been reported for snakes that the duplicated control regions and their adjacent gene segments were the access point for rearrangements, and that specifically for this group the mechanism of maintenance of duplicated control regions is the source of the structural diversity of the mitogenome^18^.

Genomes with small size, such as mitochondrial genomes, have fewer mutational targets compared to genomes with large sizes (such as the nuclear genome), so convergent evolution through homologous site mutations is expected to occur more commonly in smaller genomes^77^. Identical and completely homoplastic gene orders have previously been identified in vertebrate mitochondrial genomes; including the same architecture among one species of Amphisbaenia (Reptilia) and the architecture shared in most birds where the block *nad6, trnE* is switched in order with *cob, trnT, trnP* relative to the arrangement commonly found for vertebrates^78^ besides the architecture shared between a species Gymnophiona (Amphibia) and the architecture reported for marsupials (Mammalia) involving the WANCY region^24^.

The large number of species involved in the convergences (764) observed in this work, favors the view that convergent evolution is a general phenomenon of the vertebrate mtDNA, at least in these hotspot regions, as had been earlier predicted^8,23^. Our results support those convergences occur in two cases, (A) in which nearest neighbor tRNA genes exchange their position as is the case in convergences 4, 6, 7 and 8 and (B) in genes flanking either of the two origins of replication as occurs in convergences 1, 2, 3, 5 9, 10 and 11 reported in this study.

## Conclusion

The analysis of vertebrate mitochondrial genomes available in the database that we performed in this work identified a high error percentage in gene annotations, in addition, it shows several significant rearrangement events (especially in tRNA genes) in these organisms, contrary to what has been believed for many years. In addition, we show that the types and frequency of rearrangements in genomes behave differently between vertebrate classes and between taxonomic orders of classes. Besides, the findings from this study provide new evidence of convergence events in the gene order among vertebrate mitogenome, which could be considered as common in this species. The comparative study of hundreds of vertebrate mitogenomes provided new evidence on the evolution of this extranuclear genome, which could provide a partial explanation for some molecular adaptation processes, population biology, and lifestyle in this species group.

## Methods

### Mitochondrial genome sequences of vertebrate species

We retrieved the sequences and gene annotations of the 2831 complete vertebrate mitochondrial genomes, representing 143 taxonomic orders organized into 12 taxonomic classes (Myxini, Petromyzonti, Elasmobranchii, Holocephali, Cladistii, Actinopteri, Coelacanthi, Dipneusti, Amphibia, Reptilia, Aves, and Mammalia) that are available at the organelle genome resources database from NCBI (https://www.ncbi.nlm.nih.gov/genome/browse#!/organelles/ ) as of December 20, 2019. Mitogenomes representing strains within the same species were not included (as in the case of the mouse, *Mus muscullus*, for which there are now mitogenome sequences for at least 20 strains). A list of these species, sorted taxonomically, with the GenBank Reference IDs and reported gene rearrangements is provided in the Supplementary Table S1.

### Gene order and rearrangements rate analysis

We attempted to verify the correctness of reported gene annotations based on the methodology previously used by Prada and Boore^22^. By observational analysis using Geneious version 4.8.5^79^, the ancestral order of genes in the vertebrate mitochondrial genome postulated by different authors^1,2,43^ was compared against the genes and their annotations in the sequences obtained from GenBank. For this aim, a numerical gene order was made (from 1 to 37, considering 1 as the *trnF*, 2 as *rrnS* gene; and so on) and their gene orientation according to the position of the gene in the heavy strand (as +) and light strand (as -). For this analysis, the D-loop region was not considered due to the absence of the sequence (complete or partial), absence of annotation or presumed duplications in many of the sequences examined.

Genes that differed from the ancestral organization for vertebrates were individually extracted and different bioinformatics tools were used to corroborate whether the annotations were correct or not, tools used included alignments with the MUSCLE multiplex algorithm^80^ and NCBI-BLAST2 (https://blast.ncbi.nlm.nih.gov/Blast.cgi) in both cases the paired identity of the gene with putative rearrangement was checked against the gene whose annotation was correct from the evolutionarily closest organism with 80% sequence identity as the threshold for determining the correct orientation; MITOS web server^81^ were used to corroborate the annotation, size and orientation of tRNAs and coding genes, and tRNAscan-SE 2.0^82^ were used to detect the position of tRNA-encoding genes and confirm their orientation in mitochondrial genomes.

The gene rearrangements proportion of each Vertebrate class and/or order, by means of the following Eq. (1):

1 $$PRo: \frac{NRo}{{NE*37}}*100$$

PRo: Proportion of rearrangements for class and/or order; NRo: Number of rearrangements for orders; NE: Number of species of each order; 37: Total mitochondrial genome genes.

The gene rearrangements proportion of each Vertebrate class for gene, by means of the following Eq. (2):

2 $$PRg:\frac{NRg}{{NE}}*100$$

RRg: Proportion of rearrangements for gene; NRg: Number of rearrangements for gene; NE: Number of species of each order.

These results were compared with those obtained by the qMGR program^4^. he results generated by the analysis of gene rearrangements proportion and those of qMGR of each vertebrate order were graphed using the Heatmapper program^83^. A phylogeny was constructed from reference molecular phylogeny of the vertebrate^84^; contrasting them with those presented in this study.

### Ancestral state estimation and phylogenetic analysis

Several methodologies were used to estimate ancestral state in the vertebrate mitochondrial genome. Common interval analysis was performed using CREx^85^ for pairwise comparisons and TreeREx^86^ for genome ancestral state inference. Paired CREx comparisons of the representative mitochondrial genome of some taxonomic classes or orders against the vertebrate ancestral mitochondrial genome were performed to determine the number of minimal genome rearrangement events separating each taxonomic order from the ancestral state. The CREx and TreeREx programs use the same set of rearrangement events: transpositions, inversions, reverse transpositions and TDRL, as well as the same algorithm called common interval, however, they cannot analyze gene duplications or deletions.

Mitochondrial genomes within each vertebrate taxonomic order were aligned using the MAUVE aligner software v.2.3. progressive alignment algorithm^87^. Briefly, MAUVE involves an efficient methodology for constructing multiple whole-genome alignments regarding large-scale evolutionary events, such as rearrangement and inversion. The resulting alignments represented a mosaic of rearranged segments which were conserved among complete genomes, subsets of genomes, or unique genome segments^87,88^. Alignments were made using the following parameters: skip-refinement and seed-weight = 15, total alignment, determining local collinear blocks (LCB) and pairs of LCBs. The option of using seed families in the anchorage and linear genomes was ignored, the Mauve phylogenetic trees were used for the TreeREx analysis.

To identify gene rearrangements leading to an identical genomic architecture between species of different vertebrate lineages we used the GRIMM software (http://grimm.ucsd.edu), with parameters indicating options for circular chromosome, appropriate to analyze mitochondrial genomes; remaining parameters were settled by default. We obtained a matrix of genomic distances, comparing genomic architecture and identifying the minimum permutations required to transform one architecture to another through inversion of sintenic blocks, based in Hannenhalli and Pevzner (HP) algorithms^89^ to calculate genomic distances for multiple genomic rearrangements^90^. The identical architectures required zero permutations and were considered as convergences in gene rearrangements among different vertebrate species.

### Ethics approval and consent to participate

There was no animal experimentation undertaken in this study.

## Supplementary Information

Supplementary Information 1.

Supplementary Information 2.

Supplementary Information 3.

Supplementary Information 4.

Supplementary Information 5.
