# Supplementary material for: Comparative genomic analysis of vertebrate mitochondrial reveals a differential of rearrangements rate between taxonomic class
Source: Sci Rep. 2022 Mar 31;12:5479. doi: 10.1038/s41598-022-09512-2 (PMC8971445; doi:10.1038/s41598-022-09512-2)
Supplement: Supplementary file 1 — Supplementary Information 1. [file 41598_2022_9512_MOESM1_ESM.docx]

**Supplementary Materials**

Table S1. Analysis of gene arrangements of vertebrate mitochondrial genome. NCBI with Reference IDs shown for each species. The numeral in parentheses indicates the number of species belonging to each taxonomic group. Each gene is assigned a number 1 to 37 at the top of the Table which is then used to describe the annotated gene arrangement for each species. Each gene is transcribed left-to-right as shown except for those with a minus (-) symbol to indicate opposite orientation. The ancestral gene order has been inferred to be as postulated by [Boore ^2^](file:///C:\Users\JavaG\AppData\Local\Temp\Rar$DIa15976.9463\main_modificado%20-%20Final.docx#_ENREF_2). Highlighting indicates all deviations from that ancestral arrangement as confirmed by the bioinformatics tools used in this study, with being yellow to gene inversion-translocation, light blue to gene duplication, purple to pseudogenes and red to gene deletions.

Table S2. Mitochondrial genome rearrangement proportion in each gene by taxonomic order presenting species with rearrangements in vertebrates. (M) manual rearrangement proportion analysis and qMGR is indicated for each mitochondrial gene.
