# Supplementary material for: Comparative genomic analysis of vertebrate mitochondrial reveals a differential of rearrangements rate between taxonomic class
Source: Sci Rep. 2022 Mar 31;12:5479. doi: 10.1038/s41598-022-09512-2 (PMC8971445; doi:10.1038/s41598-022-09512-2)

Fig. S1. Parsizmonious evolutionary scenario leading to the extant gene orders in vertebrates by CREx analysis. Arrows show transcriptional orientation. tRNA-encoding genes are labeled with the one-letter code for the corresponding amino acid. (A) Actenopteri-Anguiligormes partial ancestral order, (B) Actenopteri-Pleuronectiformes ancestral order, (C) Amphibia-Anura partial ancestral, (D) Reptilia-Lacertilia-Chamaelonidae and Agamidae ancestor order, (E) Reptilia-Serpentes - Infraorder Alethinophidia ancestor, (F) Reptilia-Crocodilia ancestor order, (G) Aves ancestral order, (H) Mammalia-Marsupialia ancestor order. Each evolutionary event (reversal, transposition, and reversed translocations) is signaled to transform the ancestral genome into the derived mitochondrial genome. T he superscript indicates the taxonomic orders that have partial ancestor (ancestor with reorganizations and with ancestral order)

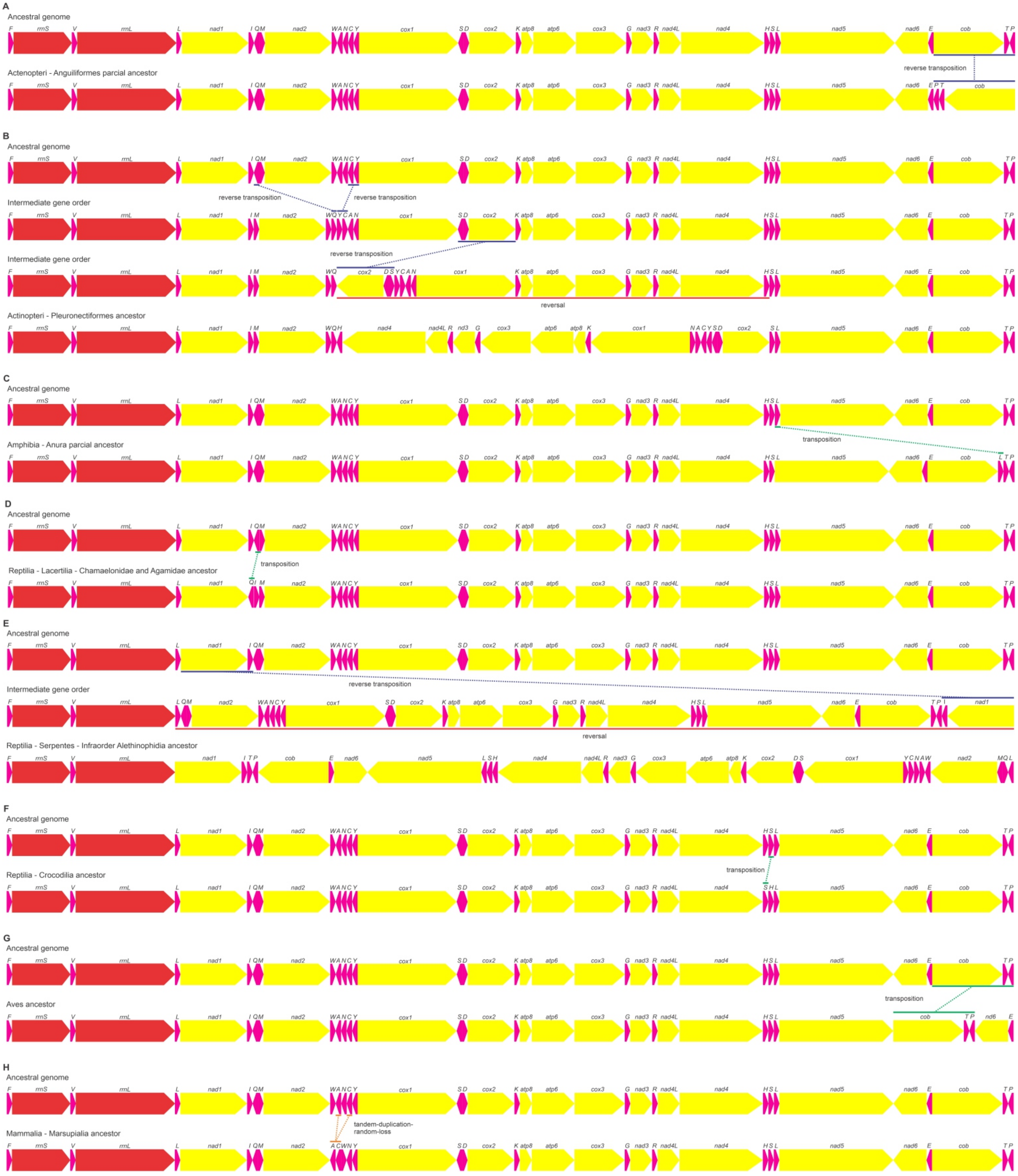

Supplement: Supplementary file 4 — Supplementary Information 4. [file 41598_2022_9512_MOESM4_ESM.pdf]
