# Supplementary material for: Comparative genomic analysis of vertebrate mitochondrial reveals a differential of rearrangements rate between taxonomic class
Source: Sci Rep. 2022 Mar 31;12:5479. doi: 10.1038/s41598-022-09512-2 (PMC8971445; doi:10.1038/s41598-022-09512-2)

Fig. S2. TreeREx analysis for vertebrate class. (A) Actinopteri - Saccopharyngiformes, (B) Actinopteri - Myctophiformes, (C) Actinopteri - Pleuronectiformes, (D) Amphibia – Anura, (E) Lacertilia - Reptilia. The rearrangements on the branches are given as Transposition, Inversion, Inverse transposition, and Tandem-Duplication-Random-Loss events (TDRLs).

A. Actinopteri - Saccopharyngiformes

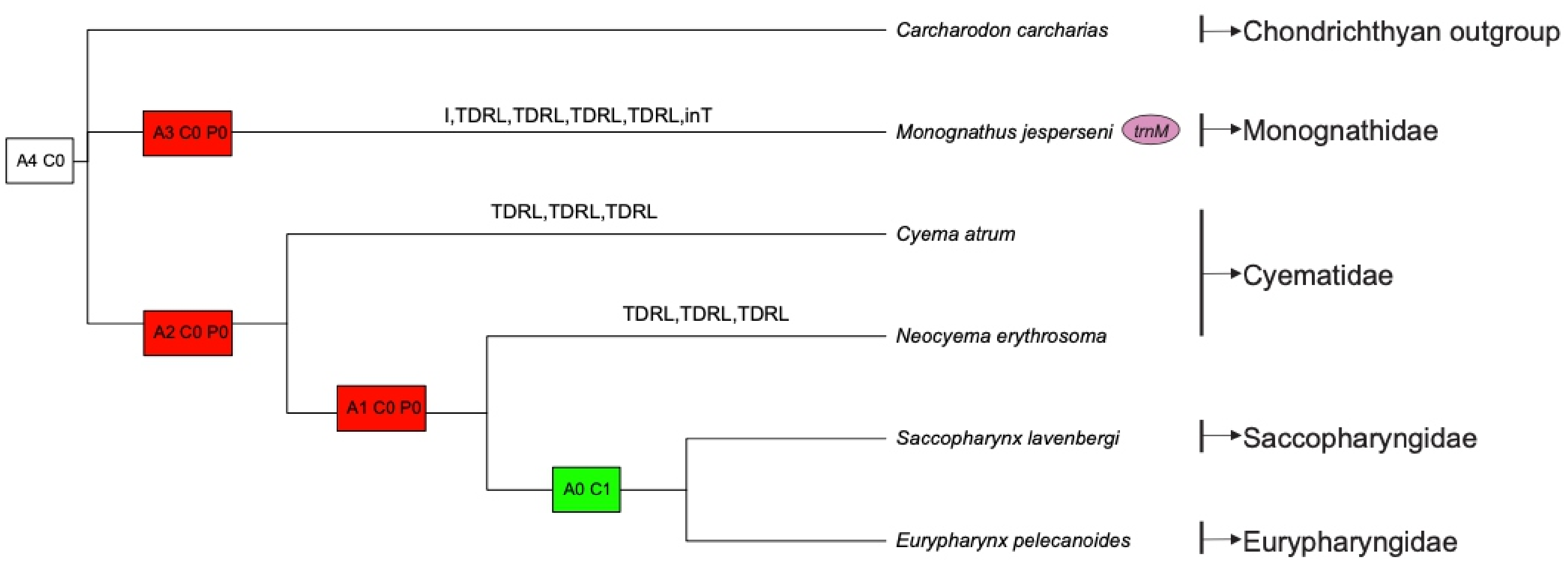

B. Actinopteri - Myctophiformes

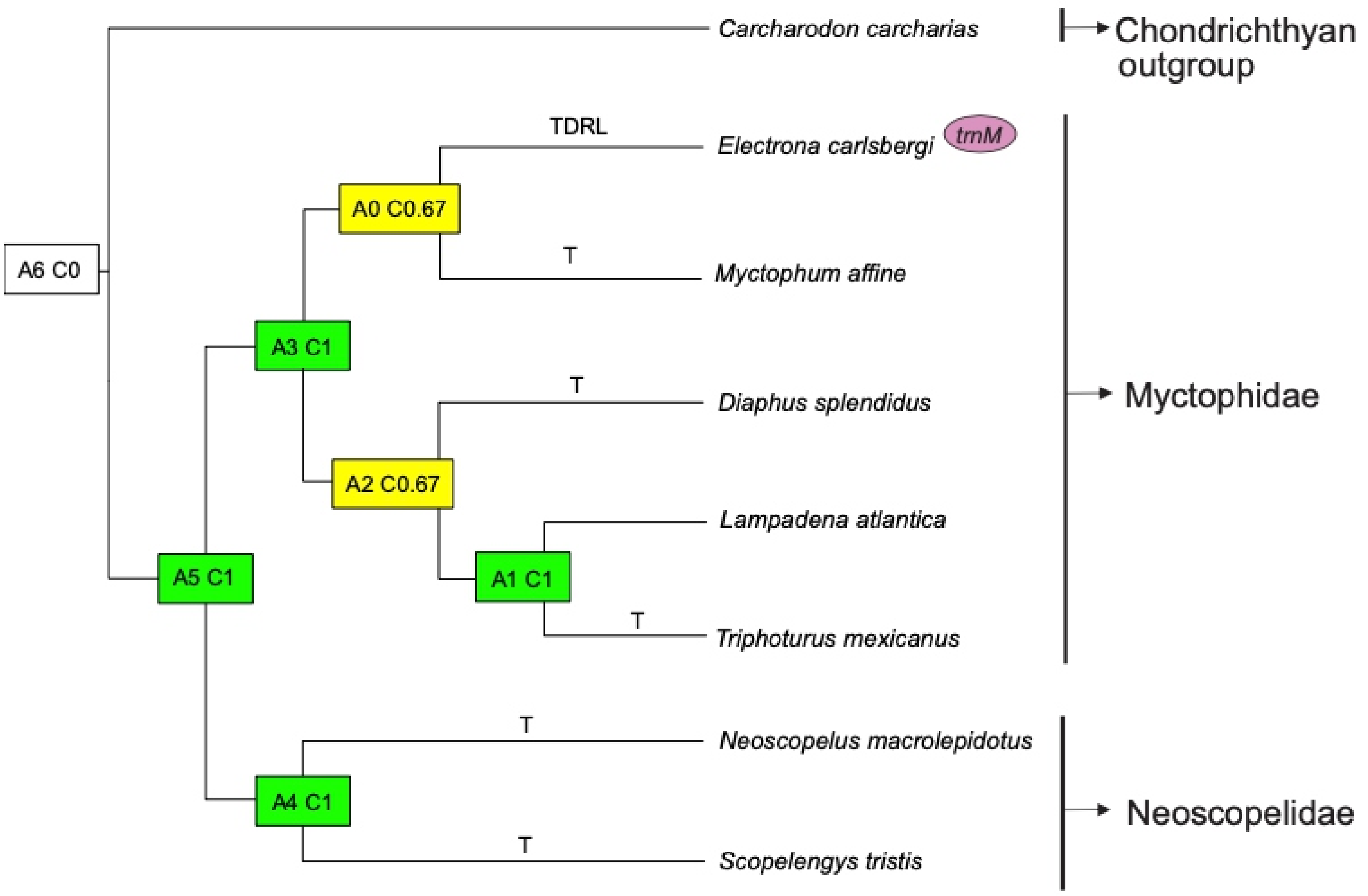

C. Actinopteri - Pleuronectiformes

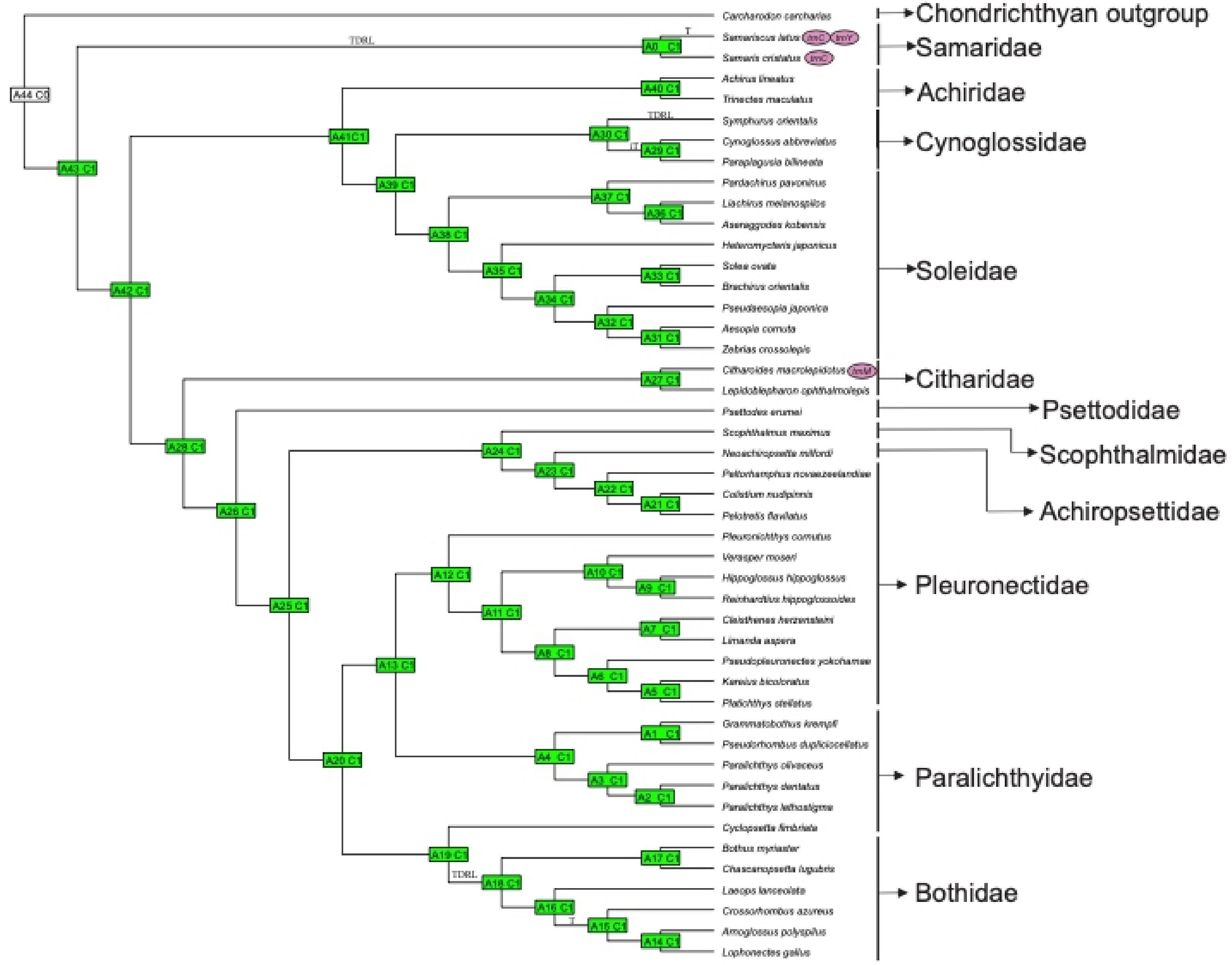

D. Amphibia - Anura

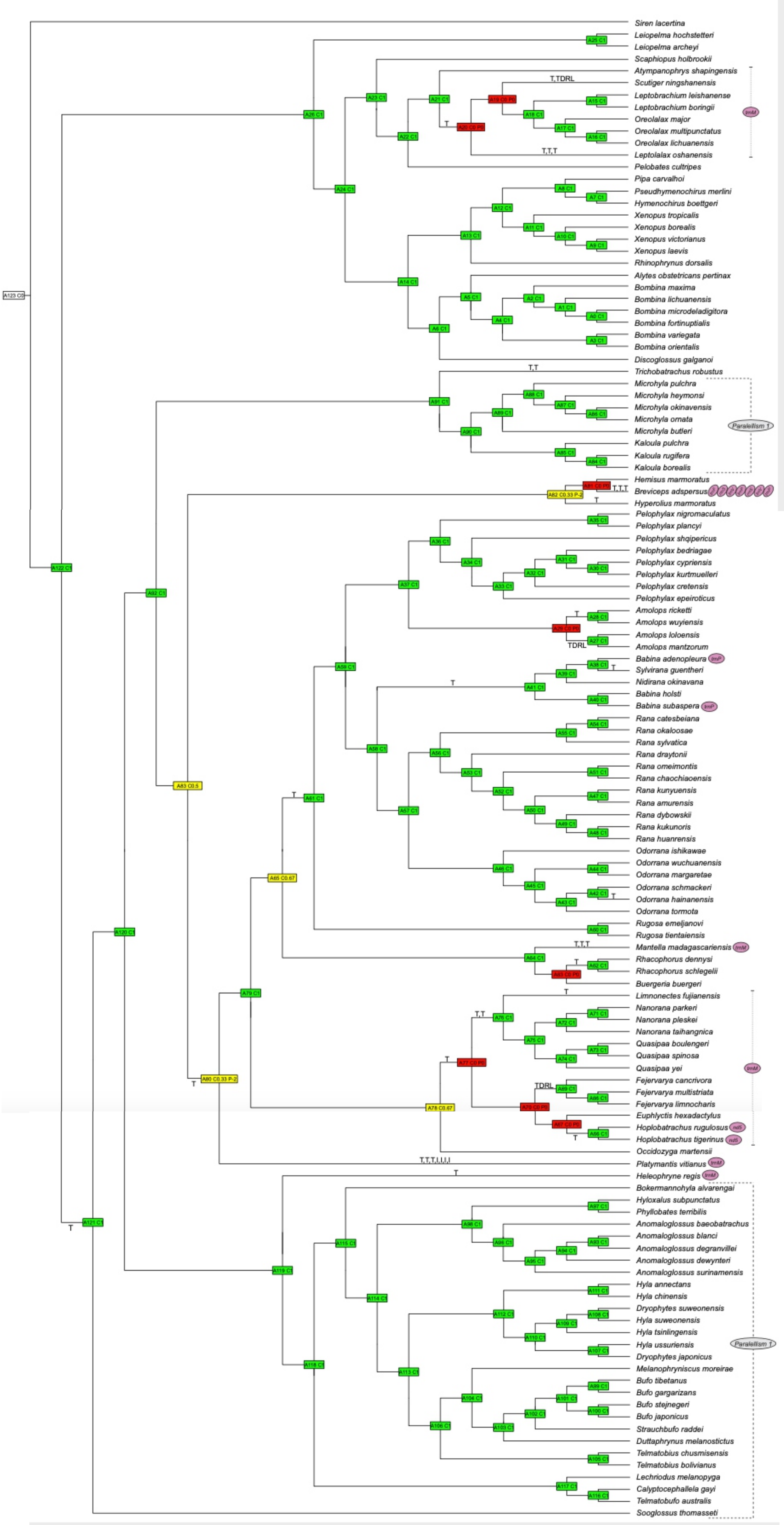

E. Reptilia - Lacertilia

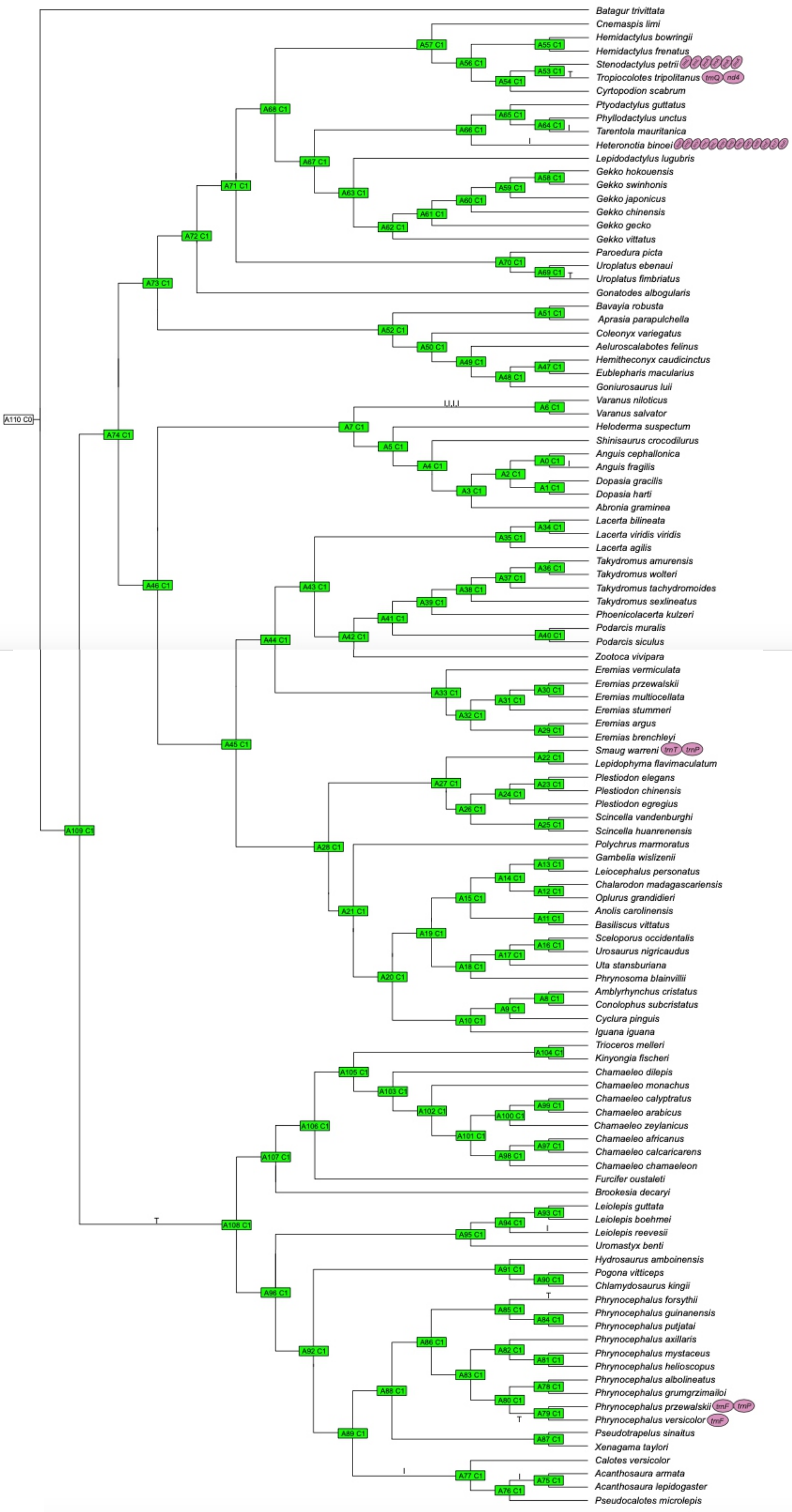

Supplement: Supplementary file 5 — Supplementary Information 5. [file 41598_2022_9512_MOESM5_ESM.pdf]
